# A high-throughput cell-based assay pipeline for the preclinical development of bacterial DsbA inhibitors as antivirulence therapeutics

**DOI:** 10.1038/s41598-021-81007-y

**Published:** 2021-01-15

**Authors:** Anthony D. Verderosa, Rabeb Dhouib, Yaoqin Hong, Taylah K. Anderson, Begoña Heras, Makrina Totsika

**Affiliations:** 1 Institute of Health and Biomedical Innovation and Centre for Immunology and Infection Control, School of Biomedical Sciences, Queensland University of Technology, Brisbane, QLD 4059 Australia; 2 Department of Biochemistry and Genetics, La Trobe Institute for Molecular Science, La Trobe University, Bundoora, Australia

**Keywords:** Antimicrobials, Bacteriology, Pathogens, Microbiology, Clinical microbiology, Drug discovery, High-throughput screening, Phenotypic screening, Oxidoreductases, Transferases, Drug discovery and development, Screening, Target validation

## Abstract

Antibiotics are failing fast, and the development pipeline remains alarmingly dry. New drug research and development is being urged by world health officials, with new antibacterials against multidrug-resistant Gram-negative pathogens as the highest priority. Antivirulence drugs, which inhibit bacterial pathogenicity factors, are a class of promising antibacterials, however, their development is stifled by lack of standardised preclinical testing akin to what guides antibiotic development. The lack of established target-specific microbiological assays amenable to high-throughput, often means that cell-based testing of virulence inhibitors is absent from the discovery (hit-to-lead) phase, only to be employed at later-stages of lead optimization. Here, we address this by establishing a pipeline of bacterial cell-based assays developed for the identification and early preclinical evaluation of DsbA inhibitors, previously identified by biophysical and biochemical assays. Inhibitors of DsbA block oxidative protein folding required for virulence factor folding in pathogens. Here we use existing *Escherichia coli* DsbA inhibitors and uropathogenic *E. coli* (UPEC) as a model pathogen, to demonstrate that the combination of a cell-based sulfotransferase assay and a motility assay (both DsbA reporter assays), modified for a higher throughput format, can provide a robust and target-specific platform for the identification and evaluation of DsbA inhibitors.

## Introduction

In 2014 the World Health Organisation (WHO) released a statement declaring antimicrobial resistance (AMR) as a public health priority that demands decisive global action^1^. Although WHO’s statement has increased AMR awareness, at the time of writing over half a decade has passed, and little progress has been made in developing effective solutions^2,3^; meanwhile, AMR rates continue to rise. The current AMR crisis demands the urgent development of effective strategies to tackle bacterial infections. One actively researched strategy is the development of antivirulence therapeutics, which have recently been gaining momentum as effective antibacterials that can circumvent the mechanisms of antibiotic resistance and eliminate or reduce resistance selection^4^. Antivirulence drugs target bacterial virulence factors and are designed to disarm pathogens, unlike conventional antibiotics which either kill or inhibit bacterial growth^5,6^. Targeting virulence factors can attenuate a pathogen’s ability to cause infection and render bacteria susceptible to the host's defence systems^7^. Consequently, virulence factors present a plethora of attractive targets for the development of new therapeutics.

Although several antivirulence drugs are currently under various stages of development, (e.g. toxin, adhesin, enzyme, secretion and quorum sensing inhibitors^6,8,9^) the potential of any antivirulence drug candidate for further clinical development relies on having established robust assays for evaluating their efficacy in vitro and in vivo^10^. While the development of antibiotics over the past several decades has benefited from standardised and comprehensive preclinical and clinical evaluation methods, the field of antivirulence drugs has had minimal guidelines for consistent testing, with only a few general guidelines reported for some types of inhibitors, e.g. for quorum sensing^10,11^. In addition, antivirulence inhibitor screening campaigns often utilise biophysical and/or biochemical assays (when the target is known), which do not allow early evaluation of inhibitor effects on bacterial cells^12^, or on cell-based virulence assays (target agnostic), which might be prone to bias by reporting non-specific inhibitor effects^11^. Here we develop a pipeline of robust cell-based assays for the in vivo evaluation of inhibitors against the DsbA antivirulence target.

In Gram-negative pathogens, the biogenesis and function of many virulence factors are intrinsically linked to the redox enzyme pair of DsbA and DsbB^13–16^. DsbA is a periplasmic oxidoreductase which catalytically introduces disulfide bonds into secreted and outer membrane proteins^17^, while its inner membrane partner DsbB reoxidises DsbA^18,19^. Intramolecular disulfide bonds are often essential for the native folding and subsequent function of multiple secreted or surface proteins, including fimbriae, flagellar motor, secretion systems, and secreted toxins^13,16^. Given that many of these proteins are *bona fide* virulence factors or form integral components of machinery for virulence factor assembly, this makes DsbA and DsbB ideal targets for the development of antivirulence drugs^13,16,20^. Recently, several classes of small molecule inhibitors of DsbA, as well as inhibitors of its cognate DsbB, have been reported, primarily through screening campaigns involving biophysical and/or biochemical assays^12,21–26^. Any in vivo assessment of promising hits was typically conducted as part of subsequent testing, often at a stage where significant efforts into the chemical elaboration of initial hits had already taken place. Incorporation of cell-based testing at an earlier stage of inhibitor screening, as conducted for DsbB and its homologue VKOR^24^, could be used to complement early hit selection by biophysical/biochemical approaches and likely save time and money, by informing which hits should be prioritised and what properties should be optimised (e.g. solubility, cell permeability, toxicity etc.).

For monitoring DsbA function in vivo, the bacterial motility assay on soft agar has been most commonly used^27–29^ and more recently this method was applied to DsbA inhibitor testing in vivo^12,30^. In many pathogens, such as uropathogenic *Escherichia coli* (UPEC), and *Salmonella enterica* serovar Typhimurium (*S.* Typhimurium), motility requires the production of functional flagella, with DsbA playing a central role in the biogenesis of these surface appendages^27,31–34^. The standard bacterial motility assay format (performed in Petri dishes) is however relatively low-throughput and requires large inhibitor quantities and manual data collection^30^, thus, limiting its utility for high-throughput inhibitor screening and testing. A second method recently utilised for DsbA inhibitor testing monitors the enzymatic activity of ASST^30^, an arylsulfate sulfotransferase encoded by several pathogens (e. g. UPEC, *S.* Typhimurium, *Klebsiella*^28,29,35,36^), which is proposed to play a role in the intracellular detoxification of phenolic substances^37–39^. ASST is a native substrate for DsbA and its homologue DsbL^40^ as it requires the formation of a disulfide bond for its correct function^41^. Consequently, ASST’s sulfotransferase activity can be used to measure DsbA activity in vivo*,* and can be monitored either in solution^40^ or using an agar-based assay^28^. Although very informative, previously used ASST assays have not been amenable to high-throughput inhibitor screening and testing. Here, we present a comprehensive pipeline of cell-based assays that provide an accurate and high throughput platform for the identification of DsbA inhibitors and their subsequent development from hits to leads, and from lead optimisation to early preclinical candidate validation.

## Results

### Establishing a high-throughput assay for in vivo monitoring of ASST enzyme activity in pathogenic bacteria

**Figure 1:**
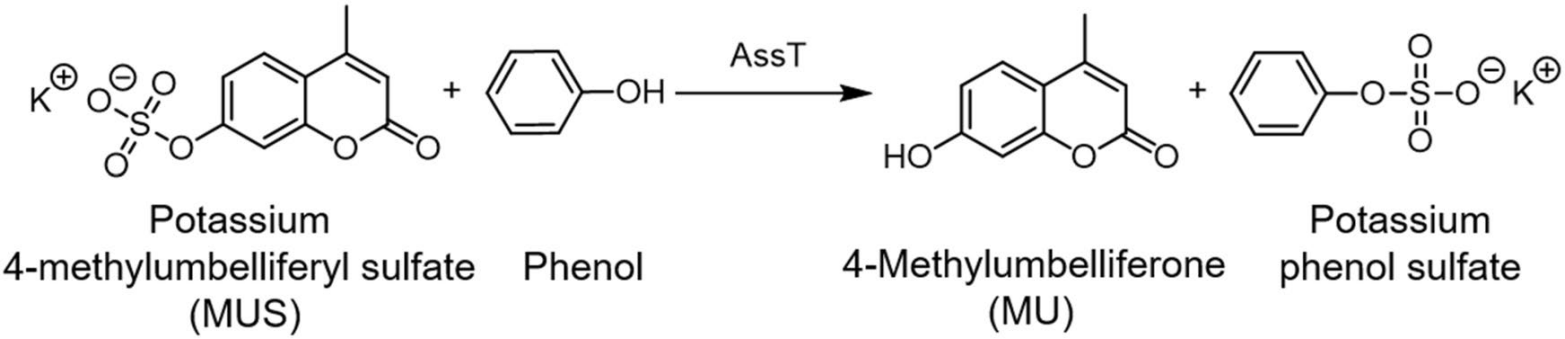
ASST catalysed conversion of MUS to MU.

**Figure 2:**
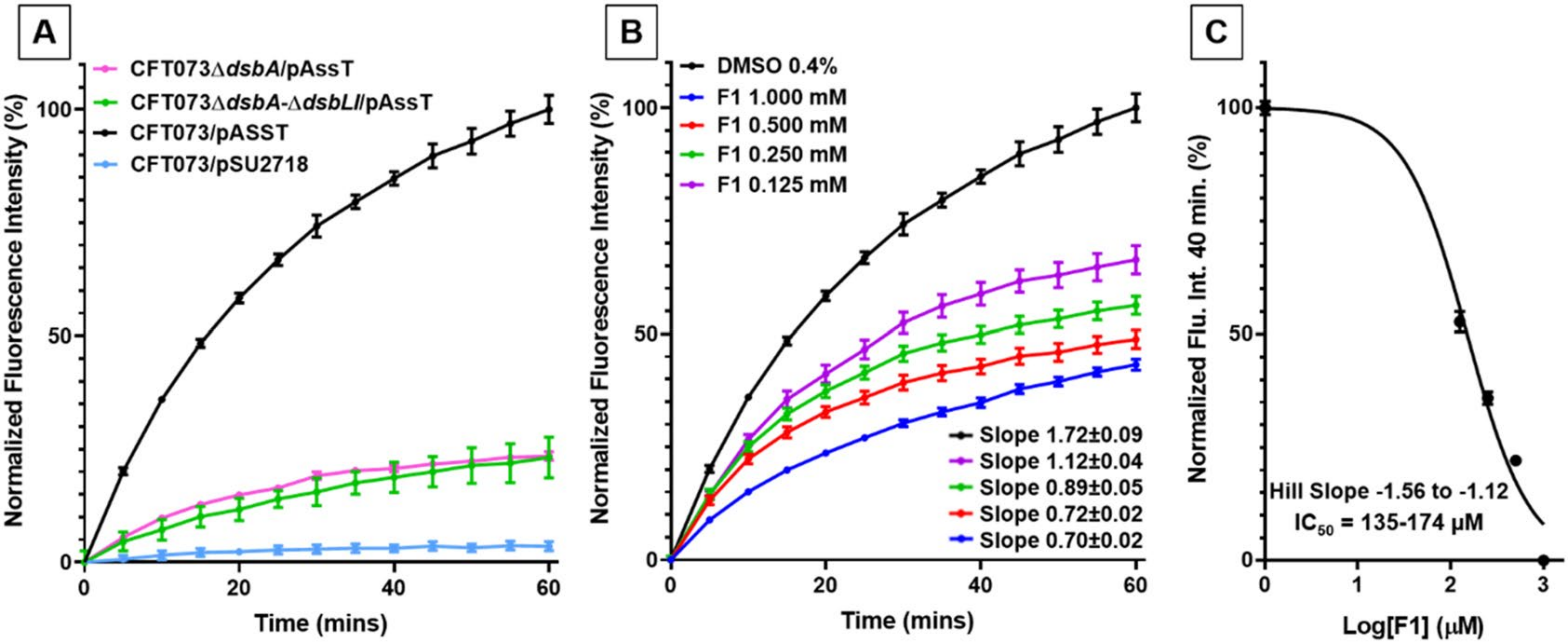
Cell-based ASST activity in UPEC CFT073 in varying concentrations of DsbA inhibitor F1. Sulfotransferase activity of (**A**) CFT073/pASST, CFT073/pSU2718 (vector control), CFT073Δ*dsbA*/pASST, and CFT073Δ*dsbA*Δ*dsbLI*/pASST cultured in 0.4% DMSO in the absence of DsbA inhibitors. (**B**) Sulfotransferase activity of CFT073/pASST cultured in the presence of F1 (1–0.125 mM) or 0.4% DMSO (vehicle control) and (**C**) corresponding F1 dose–response curve calculated at 40-min time point. F1 treated bacterial cultures were mixed with MUS and phenol and immediately monitored spectrofluorometrically (excitation 360 nm and emission 450 nm) for 60 min at room temperature (21 °C). Data are shown as normalised fluorescence intensity units (calculated by normalising the treatment data against the DMSO control (set as 100%) (**A** and **B**: slopes reported in (**B**) were calculated from the 15 to 30 min region), and normalised fluorescence intensity at 40 min (**C**) (calculated from the 40-min time point of **B**), with the mean ± SD of 3 biological replicates plotted at each time point.

Enzymatic assays are well suited to high-throughput inhibitor screening campaigns. Thus, we sought to develop a cell-based assay for monitoring the activity of the ASST enzyme, which is a known DsbA substrate in UPEC. We first determined if ASST’s sulfotransferase activity could be assayed in solution using live UPEC cells cultured in standard laboratory conditions. We specifically wanted an assay that is easy to perform using standard laboratory reagents and equipment (i.e. streamlined protocol, minimal number of steps, use of standard growth media and conditions) so that it could be easily adopted for high-throughput screening (HTS) in various settings. As the ASST gene in UPEC (*astA*) is not expressed under standard growth conditions^29^ and in order to make our assay transferrable to non ASST-encoding bacteria, we chose to produce ASST from a plasmid vector previously shown to depend on DsbA for activity in UPEC and *S. Typhimurium*^28,29,42^. The ASST overexpressing strain CFT073/pASST was cultured overnight in LB, and culture aliquots were mixed in a 96-well plate with the aryl sulfate phenolic donor, potassium 4-methylumbelliferyl sulfate (MUS) and the phenol acceptor, phenol. ASST catalysed the cleavage of the sulfate group from the non-fluorescent substrate MUS to the highly fluorescent product 4-methylumbelliferone (MU) (Fig. 1)^43^. MUS and similar substrates have been extensively used to study the activity of the ASST enzyme from various bacterial species^28,36,39,40,43,44^. A steady increase in fluorescence was observed over time for strain CFT073/pASST, but not for CFT073 carrying the empty vector control (Fig. 2A). Using these strains, we confirmed (a) the production of functional ASST which catalysed the conversion of MUS to fluorescent MU in the UPEC periplasm (where ASST and DsbA localise) and (b) determined the assay’s dynamic range. Because the chromosomal *astA* gene copy in CFT073/pSU2718 is not expressed under the assay conditions^29^, we further explored the lower limit of the assay’s dynamic range by utilising two previously characterised CFT073 mutants lacking either DsbA (CFT073Δ*dsbA*) or both DsbA and DsbL (CFT073Δ*dsbA*Δ*dsbLI*). Overexpression of ASST in these mutants (CFT073Δ*dsbA*/pASST and CFT073Δ*dsbA*Δ*dsbLI*/pASST) resulted in equally low sulfotransferase activity, displaying 23% normalised fluorescence intensity in the ASST assay (Fig. 2A). While DsbA, or one of its homologues, is required to rapidly introduce a functional disulfide bond into the ASST enzyme^43^, the disulfide bond could also form, albeit at a much slower rate, via background oxidation. This could lead to low levels of functional ASST in the cell, which would account for the activity reported for CFT073Δ*dsbA*/pASST (Fig. 2A). This 23% increase over baseline strain CFT073/pSU2718 (that does not express ASST) represents the background level of sulfotransferase activity in our assay, which likely results from environmental oxidation of ASST during overnight growth in rich media. Thus, the maximum reduction in fluorescence that can be achieved in our assay is 77%. Conducting the assay in phosphate buffer instead of media gave the same basal levels of fluorescence, suggesting that environmental oxidation of ASST could be neglected during the assay timescale (data not shown). The suitability of our assay for HTS was further evaluated by calculating the Z′-factor using the positive and negative genetic controls, CFT073/pASST and CFT073/pSU2718, respectively. Our assay has a Z′-factor of 0.89 ± 0.02 indicating excellent assay quality related to screening^45^. Taken together, these results demonstrate an effective dynamic range for the assay and confirm that it can be used to monitor DsbA-mediated ASST activity in live bacteria under standard laboratory growth conditions.

### The cell-based ASST assay offers a first high-throughput step in our DsbA inhibitor screening and development pipeline

**Figure 3:**
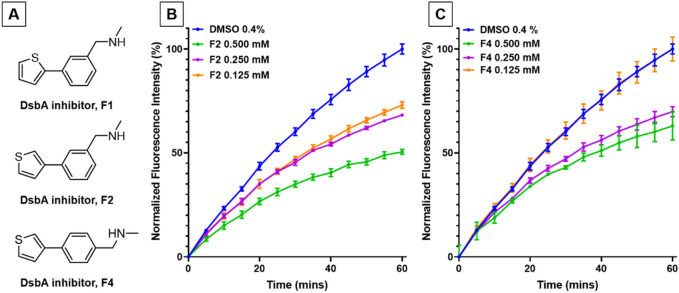
Cell-based ASST activity in UPEC CFT073 in varying concentrations of DsbA inhibitors F2 and F4. (**A**) Chemical structures of DsbA inhibitors F1, F2, and F4. Sulfotransferase activity of CFT073/pASST cultured in the presence of (**B**) F2 (0.5–0.125 mM) (**C**) F4 (0.5–0.125 mM) or 0.4% DMSO (vehicle control). Treated bacterial cultures were mixed with MUS and phenol and immediately monitored spectrofluorometrically (excitation 360 nm and emission 450 nm) for 60 min at room temperature (21 °C). Data are shown as normalised fluorescence intensity units calculated by normalising the treatment data against the DMSO control (set as 100%), with the mean ± SD of 3 biological replicates plotted at each time point.

We hypothesised that inhibition of DsbA in CFT073/pASST would result in misfolding of the ASST enzyme and loss of sulfotransferase activity. To examine this hypothesis, we repeated the ASST assay with CFT073/pASST cells treated with various concentrations of the previously described DsbA inhibitor F1 (1–0.125 mM)^42^. Sulfotransferase activity was decreased at all tested F1 concentrations (Fig. 2B), with the lowest fluorescence measured from cells cultured at 1 mM F1 (55% reduction compared to vehicle (DMSO) control). The vehicle solvent (DMSO) was found to have no effect on bacterial growth or ASST activity at the administered concentration of 0.4% (data not shown). Reduction of ASST sulfotransferase activity by F1 was dose-dependent with a half maximal inhibitory concentration (IC_50_) value in the 0.14–0.17 mM range (Fig. 2C). IC_50_ values in the mM range are indicative of modest affinity inhibitor hits that represent good candidates for synthetic optimisation. To further explore the sensitivity of the assay we also examined two other previously documented DsbA inhibitors, F2 and F4, both of which are structural isomers of F1 (Fig. 3A)^30^. Like F1, both F2 and F4 deceased sulfotransferase activity in a dose dependent manner (Fig. 3B,C). However, both inhibitors (F2 and F4) were less potent than F1 at similar concentrations, a result which is consistent with our previous in vivo findings for these compounds^30^. Taken together, these results demonstrate that DsbA inhibition results in loss of ASST sulfotransferase activity, confirming that our cell-based ASST sulfotransferase assay can be used for indirectly assessing DsbA inhibition in a high-throughput format. Furthermore, the assay is sensitive enough to distinguish small differences in inhibitor efficacy, suggesting that weaker inhibitors of DsbA can also be identified using this assay.

### The cell-based ASST assay allows target-specific testing of DsbA inhibitor activity

**Figure 4:**
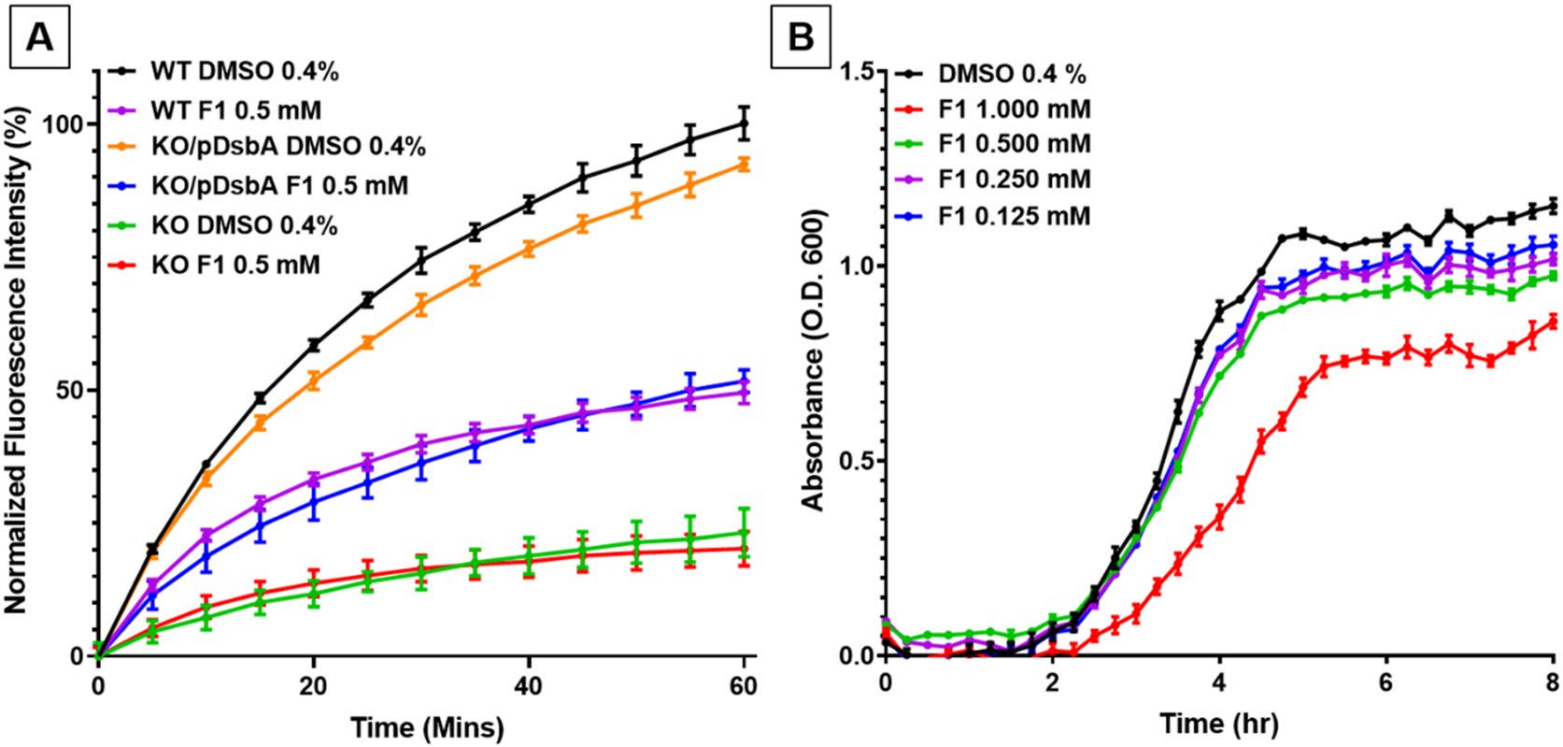
F1 inhibitor effects on DsbA cellular function and UPEC growth. (**A**) Cell-based sulfotransferase activity of CFT073/pASST (WT) grown in the presence of 0.4% DMSO or 0.5 mM F1; CFT073Δ*dsbA*Δ*dsbLI*/pASST (KO) grown in the presence of 0.4% DMSO or 0.5 mM F1; and CFT073Δ*dsbA*Δ*dsbLI*/pASST/pEcDsbA (KO/pDsbA) grown in the presence of 0.4% DMSO or 0.5 mM F1. Treated bacterial cultures were mixed with MUS and phenol and immediately monitored spectrofluorometrically for 60 min at room temperature (21 °C). (**B**) Growth curves of CFT073/pASST cultured in LB medium containing F1 (1–0.125 mM) or 0.4% DMSO (vehicle control) and monitored spectrophotometrically (Optical Density (O.D.) at 600 nm) for at least 8 h at 37 °C. Data are normalised fluorescence intensity units (calculated by normalising the treatment data against the DMSO control (set as 100%) (**A**) or absorbance at 600 nm (**B**), with mean ± SD of 3 biological replicates plotted at each time point.

With assay protocol and conditions established, we next sought to confirm the specificity of our ASST assay for the DsbA target. To investigate this, we utilised the previously described strain CFT073Δ*dsbA*Δ*dsbLI*/pASST. This strain lacks both DsbA homologues and showed decreased fluorescence compared to the wild-type strain (CFT073/pASST). *In trans* complementation with DsbA fully restored the mutant’s fluorescence back to wild-type levels (Fig. 4A), confirming that in our assay DsbA is required for the production of functional ASST enzyme. In addition, both the control strain CFT073/pASST (WT) and the complemented mutant CFT073Δ*dsbA*Δ*dsbLI*/pASST/pEcDsbA (KO/pDsbA) were equally attenuated for ASST function when treated with 0.5 mM F1 inhibitor (Fig. 4A). In contrast, the mutant CFT073Δ*dsbA*Δ*dsbLI*/pASST (KO) was unresponsive to F1 treatment, and its fluorescence profile remained unaltered upon treatment with 0.5 mM F1 or with DMSO (Fig. 4A). These results indicate that our assay can identify inhibitors that target DsbA.

### Adding a growth analysis step prior to assessing sulfotransferase activity in the cell-based ASST assay simultaneously screens for inhibitor effects on DsbA cellular function and bacterial growth

DsbA is not required for UPEC CFT073 growth in rich media and standard laboratory culture conditions^29^. As such, inhibitors specific to DsbA would be predicted to have no effect on CFT073 growth under these conditions. On the other hand, large libraries of low affinity compounds, such as those typically used in early inhibitor screens, could contain several compounds with bacterial growth toxicity. To incorporate growth testing as part of our high-throughput cell-based ASST assay, CFT073/pASST growth was continuously monitored (step 1) during culture for the preparation of live-cell samples for sulfotransferase activity testing (step 2). Testing F1 in the growth analysis step of the sulfotransferase assay, revealed that under these conditions CFT073/pASST growth was slightly reduced in the presence of 1 mM F1, with no growth defects observed at lower F1 concentrations (0.5–0.125 mM) (Fig. 4B). We have previously examined the effects of F1 on the growth of wild type UPEC CFT073 and found no significant inhibition of growth at concentrations up to 1 mM. However, the ASST assay utilises a CFT073 strain which is overexpressing ASST (CFT073/pASST) that is subjected to growth conditions that differ from those used in our original study (microaerobic plate format in this study versus aerobic in tubes in our previous study)^30^. In addition to uncovering this small growth defect at high F1 concentrations, incorporating the growth step in our assay allowed us to account for any potential reduction in viable cells present in culture samples tested for sulfotransferase activity. Having an accurate O.D. 600 nm reading at the time of culture collection, ensured that all samples tested in the ASST assay could be easily adjusted to contain the same number of live cells, which was confirmed by plating for viable CFU (3–4 × 10^8^ CFU/mL). These results demonstrate that adding a growth analysis step to the cell-based sulfotransferase enzyme assay allows growth related inhibitor effects to be identified and adjusted prior to downstream inhibitor testing.

### Establishing a plate-reader cell-based assay for monitoring bacterial motility over time

**Figure 5:**
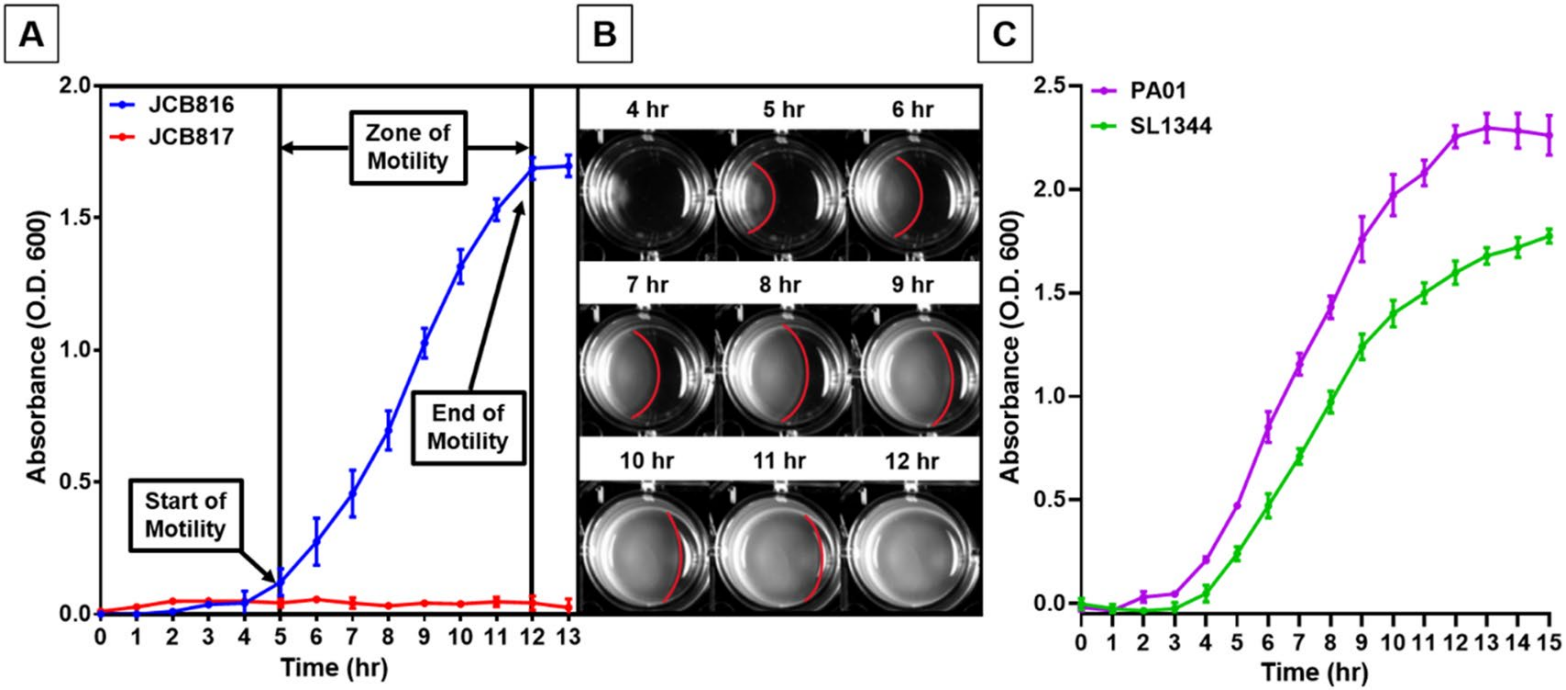
Absorbance-based monitoring of bacterial motility. (**A**) Motility curves of *E. coli* JCB816 and *E. coli* Δ*dsbA* JCB817 monitored spectrophotometrically during incubation on soft LB agar at 37 °C over 13 h. (**B**) Digital images tracking the swimming motility of *E. coli *JCB816 on soft LB agar in a 24-well plate. *E. coli *was inoculated at the left edge of each well, and by 12 h incubation at 37 °C the zone of motility (boundary marked in red) had reached the opposite edge of the well. (**C**) Motility curves of *P. aeruginosa *(PAO1) and *S. *Typhimurium (SL1344) monitored spectrophotometrically during incubation on soft LB agar at 37 °C over 15 h. Data are shown as absorbance at 600 nm (**A** and **C**), with mean ± SD of 3 biological replicates plotted at each time point.

Screening for DsbA inhibitors using cell-based assays, requires reporter phenotypes that are mediated by DsbA substrates proteins, such as ASST. Using such an approach for inhibitor screening, unavoidably could lead to hits that either target DsbA (our intended target) or the reporter protein itself (e.g. ASST). Including genetic controls can control for this, as demonstrated above for inhibitor F1 and our DsbA knock-out mutants, however, in a high-throughput screen of thousands of compounds this would require a parallel screen to be conducted with the control strain. While feasible this would not present a cost-effective design. Instead, most drug screening approaches combine orthogonal techniques for hit validation. Here we have modified a second cell-based DsbA reporter assay that relies on a different reporter substrate (the flagella motor protein FlgI) and can be used as a second stage screen of the hits identified by the high-throughput ASST assay. This not only ensures target specificity for identified hits but also presents a standalone medium-throughput assay for evaluating compounds further elaborated and developed as leads. As bacterial motility assays are the most common method of assessing DsbA inhibitor efficacy, we first sought to develop a motility assay which would circumvent the limitations of the standard assay format and provide a platform which could be employed directly after a HTS campaign. We adapted a microtiter plate-based assay which had previously been reported for screening antimicrobial compounds using bacterial motility^46^. We first confirmed that bacterial swimming motility could be accurately monitored spectrophotometrically. As bacteria radially migrated through the soft agar, a zone of motility corresponding to an increase in absorbance at 600 nm was observed, and a motility curve could be generated over time (Fig. 5A,B). Using this method, the start, end, and zone of motility of several test species (*E. coli* (JCB816), Fig. 5A; *Pseudomonas aeruginosa* (PAO1) and *S. Typhimurium* (SL1344), Fig. 5C), were accurately measured under a set of specific culture conditions. In these conditions, an *E. coli* DsbA null mutant (JCB817) remained immotile throughout the assay, demonstrating the lower end of the assay’s dynamic range (Fig. 5A).

### The absorbance-based bacterial motility assay offers a second step for hit validation and further DsbA inhibitor development in our pipeline

**Figure 6:**
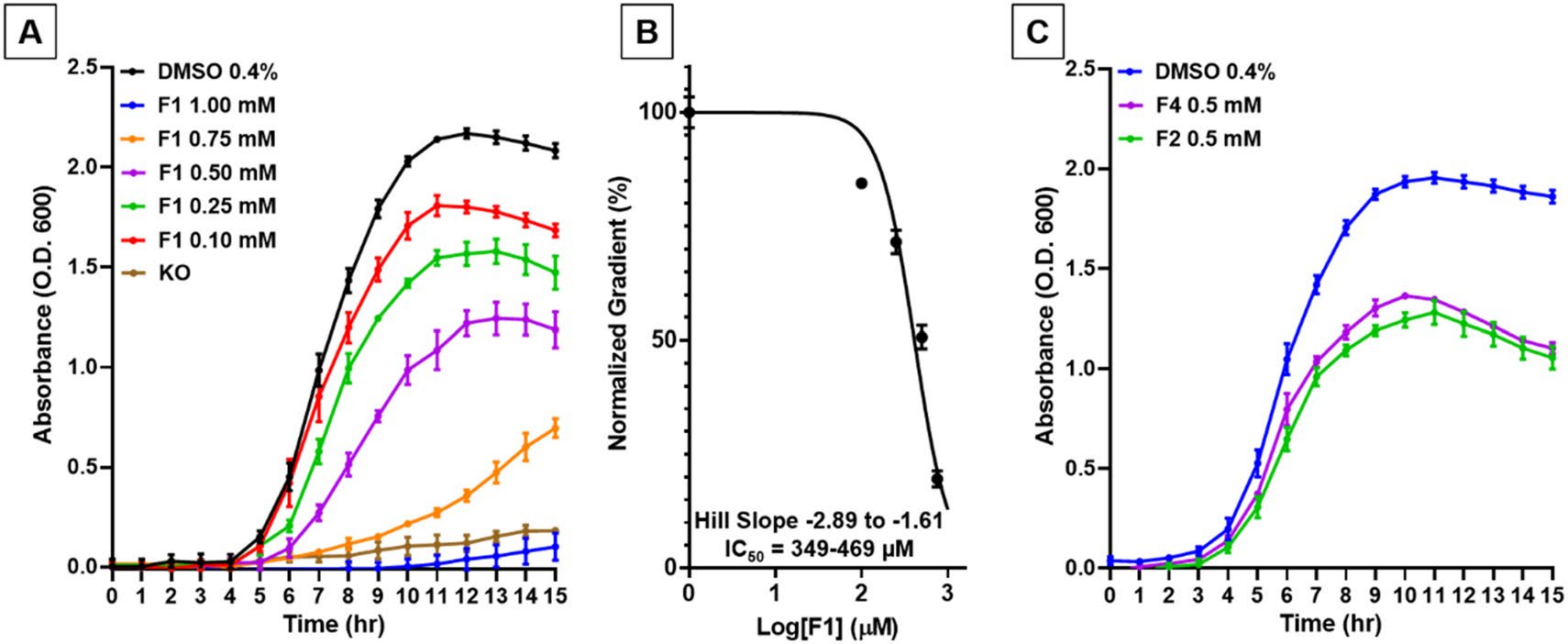
Absorbance-based UPEC motility in the presence of DsbA inhibitors. (**A**) Motility curves and (**B**) motility dose–response curve of UPEC CFT073 on LB agar (0.25%) containing DsbA inhibitor F1 (1–0.1 mM) or (**C**) DsbA inhibitors F2 (0.5 mM), F4 (0.5 mM) and 0.4% DMSO (vehicle control), monitored spectrophotometrically during incubation at 37 °C over 15 h. The immotile isogenic dsbA null strain (KO) is shown as a control (**A**). Data are shown as absorbance 600 nm (**A** and **C**), and normalised gradient (calculated from the treatment slopes of (**A**), listed in Table 1) (**B**), with the mean ± SD of 3 biological replicates plotted at each time point.

To demonstrate the value of a plate reader-based motility assay in assessing DsbA inhibitor hits, we generated motility curves for UPEC strain CFT073 in the presence and absence of DsbA inhibitor F1 (Fig. 6A), which we have previously shown to inhibit DsbA in CFT073 using the traditional petri-dish motility assay^30^. The motility of CFT073 in soft agar containing F1 at a concentration gradient (1–0.1 mM) was reduced compared to the vehicle control in a dose-dependent manner (Fig. 6A). Maximum motility inhibition was observed at 1 mM F1 were CFT073 remained immotile similar to the double *dsbA dsbL *null mutant (97% compared to DMSO control at 10 h post-inoculation) (Fig. 6A). Analysis of the longitudinal motility data revealed that both the start time and the rate of motility were directly related to F1 inhibitor concentration, with higher concentrations resulting in longer motility start times and slower motility rates (Table 1), effects that were not previously evident with the conventional motility assay methodology^27^. Furthermore, the high reproducibility of our assay allowed for even small changes in motility rate to be robustly detected (*P* < 0.0001, one-way ANOVA test) between different F1 treatment groups (Table 1). Using the calculated rate of motility for each F1 concentration, an F1 dose–response curve could be generated (Fig. 6B) with an IC_50_ for F1 calculated at the 0.35–0.47 mM range. To further confirm the assay’s suitability for validating and evaluating DsbA inhibitors, two additional inhibitors were tested. Like F1, DsbA inhibitors F2 and F4 were found to inhibit the motility of CFT073 compared to the DMSO control (Fig. 6C). Taken together, our absorbance-based motility assay proved to be of value in generating accurate and highly reproducible motility curve data that could be used to identify and characterise early hits from DsbA inhibitor screening campaigns, such as inhibitors F1, F2, and F4^12^. Moreover, this motility assay format (24-well plate) required almost 29-fold less inhibitor than the standard petri-dish assay (0.14 mg/well versus 4 mg/petri-dish) and used an automated data collection pipeline that markedly reduced the assay’s hands-on time.

**Table 1:** Motility curve parameters for UPEC CFT073 in the presence of varying concentrations of DsbA inhibitor F1.

F1 (mM)	Start of motility (h)	End of motility (h)	Rate of motility (slope)^a^	Inhibition of motility (%)^b^
1.00	11	n/d	0.02 ± 0.007	97
0.75	7	n/d	0.09 ± 0.013	89
0.50	6	12	0.21 ± 0.02	51
0.25	5	11	0.28 ± 0.02	30
0.10	5	11	0.33 ± 0.01	17
0.00	5	11	0.38 ± 0.03	n/a

^a^Motility rates shown as mean slope value ± S.D. from four biological replicates. Group means were compared using the one-way ANOVA test (*P* < 0.0001).

^b^Compared to vehicle control (DMSO 0.4%) and determined using data from the 10-h time point.

n/d = not determined.

## Discussion

Antimicrobial drug development typically starts with screening large fragment or compound libraries to identify initial hits and the subsequent chemical elaboration of different hit series. Such screening campaigns represent a big investment, in terms of time and resources, both for the industry and for the academic lab. Success relies heavily on the use of well-established, accurate reporter assays that can identify hits with some degree of target-specificity and are amenable to high-throughput testing of several thousands of compounds at once. For antibiotics, such testing is now considered routine and follows global standards and guidelines^47,48^. For non-traditional antibacterials, however, which are currently being actively explored as viable solutions to the pressing problem of AMR, consistency in drug testing and reporting is far from achieved. For antivirulence drugs in particular, a major challenge lies in standardising preclinical testing for a largely diverse set of targets that potentially mediate multiple different phenotypes in bacterial pathogens. Measuring virulence target inhibition reliably and at large-scale is often difficult when using microbiological assays, so when the target is known, inhibitor screening and early evaluation typically relies on biochemical/biophysical approaches. This is the case for DsbA inhibitors that have been reported to date, with hits from several chemical classes having been identified as part of fragment-based screening campaigns primarily using saturation transfer difference NMR spectroscopy^12,21–23^. Later-stage microbiological evaluation has validated some but not all hits, and in some cases, even chemically elaborated analogues have failed to show activity in cell-based assays^12,30^. In this study, we have optimised two cell-based assays previously used to monitor DsbA function in vivo for an accurate, streamlined, and high-throughput testing pipeline of DsbA inhibitors. When combined, these assays could support DsbA inhibitor development from hit identification to lead optimisation and preclinical candidate validation.

For developing a high throughput assay, we chose to use a read-out that is a native virulence substrate of DsbA in UPEC. ASST is a large periplasmic enzyme encoded by UPEC and other intestinal bacteria^30^ that was reported to be upregulated in the urine of UPEC-infected mice^49,50^, but was not required for colonisation of the murine bladder^30^. The gene encoding ASST (*astA*) is found in a tri-cistronic operon with the *dsbL* and *dsbI* genes, which encode an accessory redox protein pair in UPEC with specificity for ASST^40,43,51^, although the DsbA and DsbB redox pair was also shown to functionally fold ASST^28,30^. The ASST activity assay was previously performed in liquid medium using bacterial cell lysates^40^ or on solid medium using whole live cells^28^. To evaluate DsbA inhibitors, we have previously utilised the solid medium cell-based method to successfully quantify DsbA inhibition of ASST activity^30^. Despite this being an accurate cell-based assay, its petri-dish format has several limiting factors, which are preventing its use in inhibitor screens: (i) a relatively low-throughput capacity, (ii) the requirement of high inhibitor quantities, and (iii) manual data collection by endpoint imaging^28,30^. The modified ASST sulfotransferase enzyme assay presented here operates on the same principle, yet its application is quite different. The assay is conducted in liquid media using live UPEC cells treated with minimal quantities of DsbA inhibitor, and the activity of ASST is assessed in an automated fashion by monitoring the MUS-phenol sulfotransferase reaction spectrofluorometrically in real-time (rather than as an endpoint^30^). In addition, conducting the enzyme assay in liquid medium significantly increased scalability while drastically reduced reaction volumes and the amount of substrate and inhibitor needed. In fact, by performing the assay in 96-well plates the amount of substrate and inhibitor used was reduced by 100-fold compared to previous assay methodology^30^. While we showcased scalability by conducting the assay in a 96-well format, the assay can be additionally downscaled to suit a 384-well plate, which would further reduce the amount of substrate and inhibitor required (1000-fold reduction over the previous method^30^).

Our HTS ASST enzyme assay not only functions as a method for identifying DsbA inhibitors, but it could potentially be utilised for the identification of inhibitors for other targets, as it monitors both bacterial growth and sulfotransferase activity. Sulfotransferase enzymes are widespread in pathogenic bacteria (e.g. *Klebsiella* sp., *S.* Typhimurium, *Mycobacterium tuberculosis*)^35,38,52^ and eukaryotic cells, where they play a pivotal role in the detoxification of xenobiotics, drugs, and other endogenous compounds^53^. Most importantly, sulfotransferases have been linked to several disease states (e.g. cancer and infection) which has prompted studies into their specific functions^54–56^ and search for selective inhibitors^57,58^. However, very few HTS methods are available for identifying sulfotransferase inhibitors^59^. Our HTS ASST assay could be optimised for use with different sulfotransferase enzymes thus facilitating the development of selective sulfotransferase inhibitors. However, for the purposes of a DsbA targeted inhibitor screen, we aimed to ensure that our assay could differentiate between inhibitors targeting DsbA vs. growth and/or ASST. To identify and exclude potential growth inhibitors we added a bacterial growth analysis step (prior to measuring ASST activity and during bacterial treatment with inhibitors), which required no additional inhibitor, and is also easily scalable to fit the 384-well format, a benefit for future fragment-based drug design approaches. Identifying inhibitors that block ASST’s sulfotransferase activity without inhibiting DsbA required the use of genetic controls. While our ASST assay can be easily modified to run in various strain backgrounds in parallel (as demonstrated by the use of various knock-out mutants), doing so in a compound screening scenario is not ideal as it would significantly increase compound quantity requirements. As such, controlling for hit target specificity by this approach was not considered cost-effective. Instead we opted for an orthogonal technique to confirm hits from our primary screen, an approach commonly used in drug discovery campaigns. The development of a second cell-based assay that could be run subsequently to the ASST assay and was based on a different DsbA reporter phenotype (bacterial motility) presented with several added advantages.

Flagella-mediated bacterial motility is a well-established reporter phenotype for DsbA activity and thus the standard petri-dish soft agar motility assay has been successfully used to evaluate DsbA inhibitors in vivo^12,30^. However, the current format of the bacterial motility assay in petri-dishes has the same limitations of relatively low-throughput capacity, high inhibitor quantity requirement and manually intensive data acquisition of other petri-dish based assays. Our modified plate-reader motility assay utilises the same soft agar methodology, however, instead of relying on incremental images and manual measurements of motility zones, it uses a fully-automated system (plate-reader absorbance measurements) and requires no human intervention throughout the assay period making it less labour-intensive and less prone to bias or human error in motility assessments. In addition, automating the assay ensures conditions (e.g. temperature) are better controlled and can remain constant from start to finish. An important improvement was in that downscaling the assay from a petri-dish to a multi-well plate format, drastically reduced the quantity of inhibitor required (30-fold reduction in 24-well plate and 60-fold reduction in 48-well plate, compared to previous method^30^). While others have demonstrated that an agar-based motility assay can be performed in 96-well or even a 384-well plate^46,60^, these assays require specific and/or specialised equipment not typically present in the average research lab or do not allow for small differences in motility inhibition to be accurately observed and assessed. For these reasons, our absorbance-based motility assay is better suited to inhibitor confirmation and evaluation post-hit discovery.

In conclusion, our study describes the establishment of a microbiological assay pipeline that can support DsbA inhibitor development all the way from screening to early preclinical candidate validation. In addition, our assays are also well suited for screening and evaluating other types of inhibitors (e.g. for flagella components, motility regulators, quorum sensing, sulfotransferase activity), and may find multiply uses in other inhibitor screening projects. Importantly, we hope our study will serve as a paradigm for the development of similarly accurate, easy to perform, and high throughput cell-based assays that can advance the discovery and preclinical development of other antivirulence drugs that could offer future solutions to curbing the AMR crisis.

## Methods

### Bacterial strains, plasmids, and culture conditions

All bacterial strains utilised in this study (Table 2) were routinely cultured at 37 °C in liquid or on solid lysogeny broth (LB-Lennox) medium supplemented, when required, with chloramphenicol (34 µg/mL) or ampicillin (100 µg/mL), or both. CFT073 mutants were constructed previously using λ-red-mediated homologous recombination as described elsewhere^29,61^. Plasmids pASST^28^, pEcDsbA^29^, and pSU2718 were routinely transformed into strains using electroporation.

**Table 2:** Table of bacterial strains and plasmids.

Strains and plasmids	Description	Reference
*E. coli* JCB816	(JCB570 λ*malFlacZ-102*) [JCB570 is MC1000 *phoR-*, *zih12*::Tn*10tetR*	^63^
*E. coli* JCB817	(JCB571 *λmalFlacZ-102*) [JCB571 is MC1000 *phoR-*, *zih12*::Tn*10tetR*, *dsbA*::kan1	^63^
*Pseudomonas aeruginosa* (PAO1)	*Pseudomonas aeruginosa strains PAO1 (HER-1018; ATCC BAA-47)*	^64,65^
*Salmonella enterica* serovar Typhimurium (SL1344)		^66,67^
*E. coli* CFT073	UPEC isolate (O6:K2:H1)	^68^
CFT073Δ*dsbA*Δ*dsbLI*	*CFT073*Δ*dsbA::*FRTΔ*dsbLI::*FRT	^29^
CFT073/pASST		This study
CFT073/pSU2718		This study
CFT073Δ*dsbA*Δ*dsbLI*/pASST		This study
CFT073Δ*dsbA*Δ*dsbLI*/pASST/pEcDsbA		This Study
Plasmids		
pASST	*astA* gene in pSU2718; Cm^r^	^28^
pEcDsbA	*dsbA* gene in pUC19; Ap^r^	^29^

### Chemicals and stock solutions

Chloramphenicol, ampicillin, phenol, and MUS were purchased from Sigma-Aldrich (Australia), F1 and F2 were purchased from Thermo Fisher Scientific (Australia), and F4 was purchased from Synthesis Med Chem (Australia). MUS (10 mM) and phenol (50 mM) solutions were prepared in sodium chloride (0.9%), and F1 (250 mM) solution was prepared in dimethyl sulfoxide (DMSO). All stock solutions were stored in the absence of light at − 20 °C. Working solutions were prepared in LB-Lennox and were used on the same day.

### Absorbance-based bacterial motility assay

**Figure 7:**
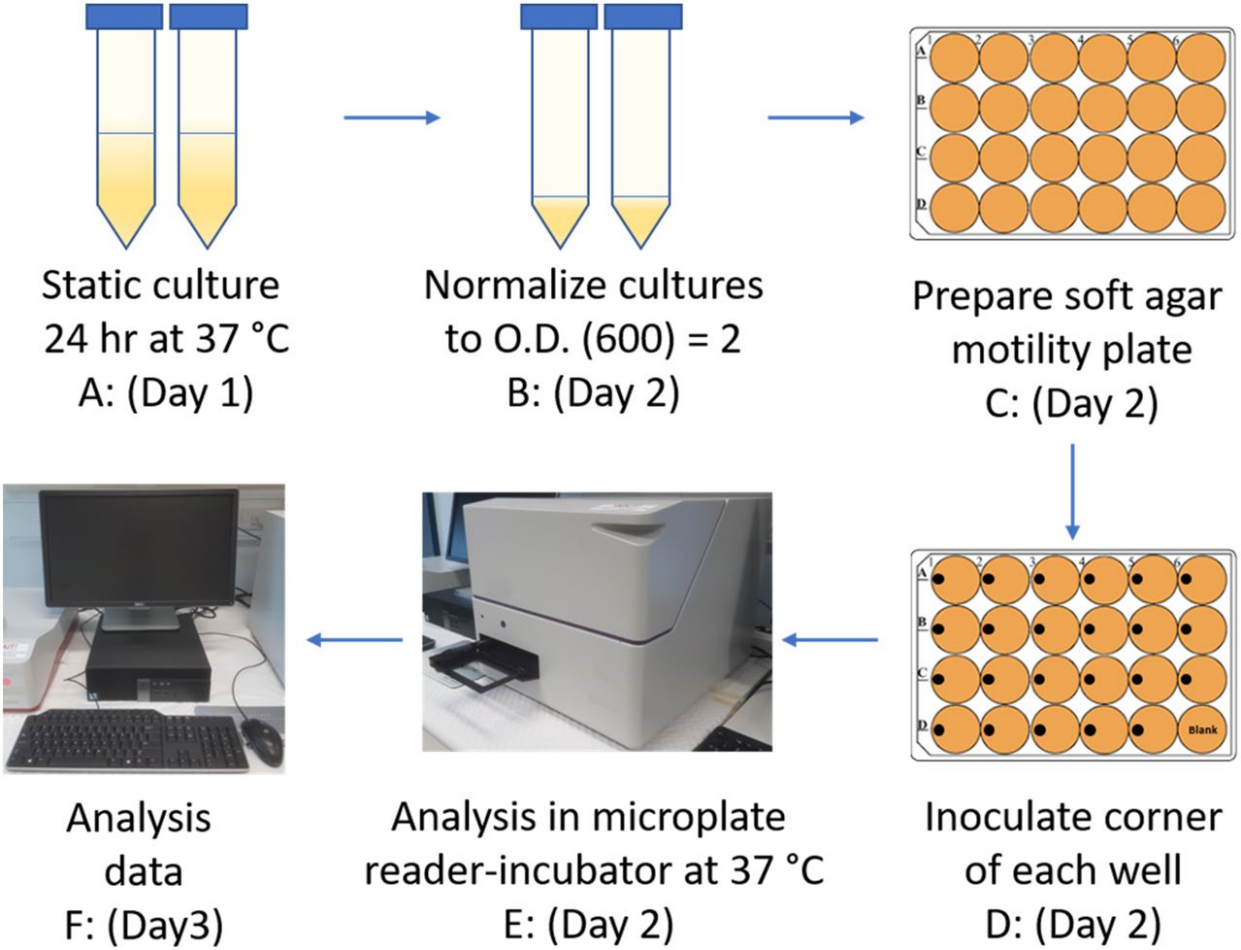
Overview of absorbance-based bacterial motility assay. (**A**) Bacterial strains were cultured statically in LB-Lennox media for 24 h. (**B**) Overnight cultures were normalized to an O.D. 600 nm of 2. (**C**) 24-well motility plates were prepared by pipetting 700 µL of warm (55 °C) soft LB agar (0.25%) in each well supplemented with F1 inhibitor or vehicle control (DMSO). The soft agar was allowed to solidify at room temperatures for at least 2 h. (**D**) 1 µL of bacterial culture fixed at O.D. 600 nm = 2 was inoculated onto the surface of triplicate soft agar wells by depositing the inoculum at the left edge of the well (with care not to penetrate the agar). The inoculum was allowed to dry onto the agar for 20 min at room temperature before the plate was covered with a plastic lid. (**E**) Bacterial swimming motility was monitored spectrophotometrically for 15-h in a plate reader (BMG Australia) at 37 °C (using orbital, spiral, or matrix averaging to ensure optimal well coverage). (**F**) Data acquisition and analysis.

Bacterial strains were grown by static 24-h culture in LB-Lennox media at 37 °C. Cultures were normalized to an O.D. 600 nm of 2 (~ 2 × 10^9^ CFU/mL) using a spectrophotometer. The multi-well soft agar plates were prepared by adding a volume of 700 µL (24-well) or 350 µL (48-well) of soft LB-Lennox agar (0.25% [wt/vol]), containing either DMSO (0.4%, vehicle control) or inhibitors F1, F2 or F4 at various concentrations (1–0.1 mM), to each well of the plate. The soft agar was allowed to solidify for at least 2 h at room temperature (21 °C), before being inoculated in the left-hand corner of each well with 1 µL of normalized culture (~ 2 × 10^9^ CFU/mL). Inoculated plates were incubated at room temperature for 20 min to allow the inoculum to dry. The zone of motility was measured by incubating plates at 37 °C in a CLARIOstar plate reader (BMG, Australia) programmed to measure absorbance (O.D. 600 nm) at each hour over 15 h (Fig. 7). Absorbance measurements were made using the inbuilt spiral averaging function with orbital averaging producing similar results (data not shown). Instrument data were normalized (with DMSO vehicle control data set at 100%) and plotted using GraphPad Prism 8. Mean motility values were calculated from 3 biological replicates of each strain tested under each specific condition. The start and end of motility were estimated from motility curves and were defined as the beginning and endpoints, respectively, of the exponential phase (zone of motility). The slope of each zone of motility was calculated in Excel and group means were compared for statistical differences by one-way ANOVA (*p* < 0.05) in GraphPad Prism 8. F1 dose–response curves were generated using the motility slopes, and the corresponding IC_50_ value was calculated by applying a non-linear regression (curve fit) Y = 100/(1 + 10^((LogIC50-X) × HillSlope))).

### Cell-based ASST sulfotransferase enzyme assay

**Figure 8:**
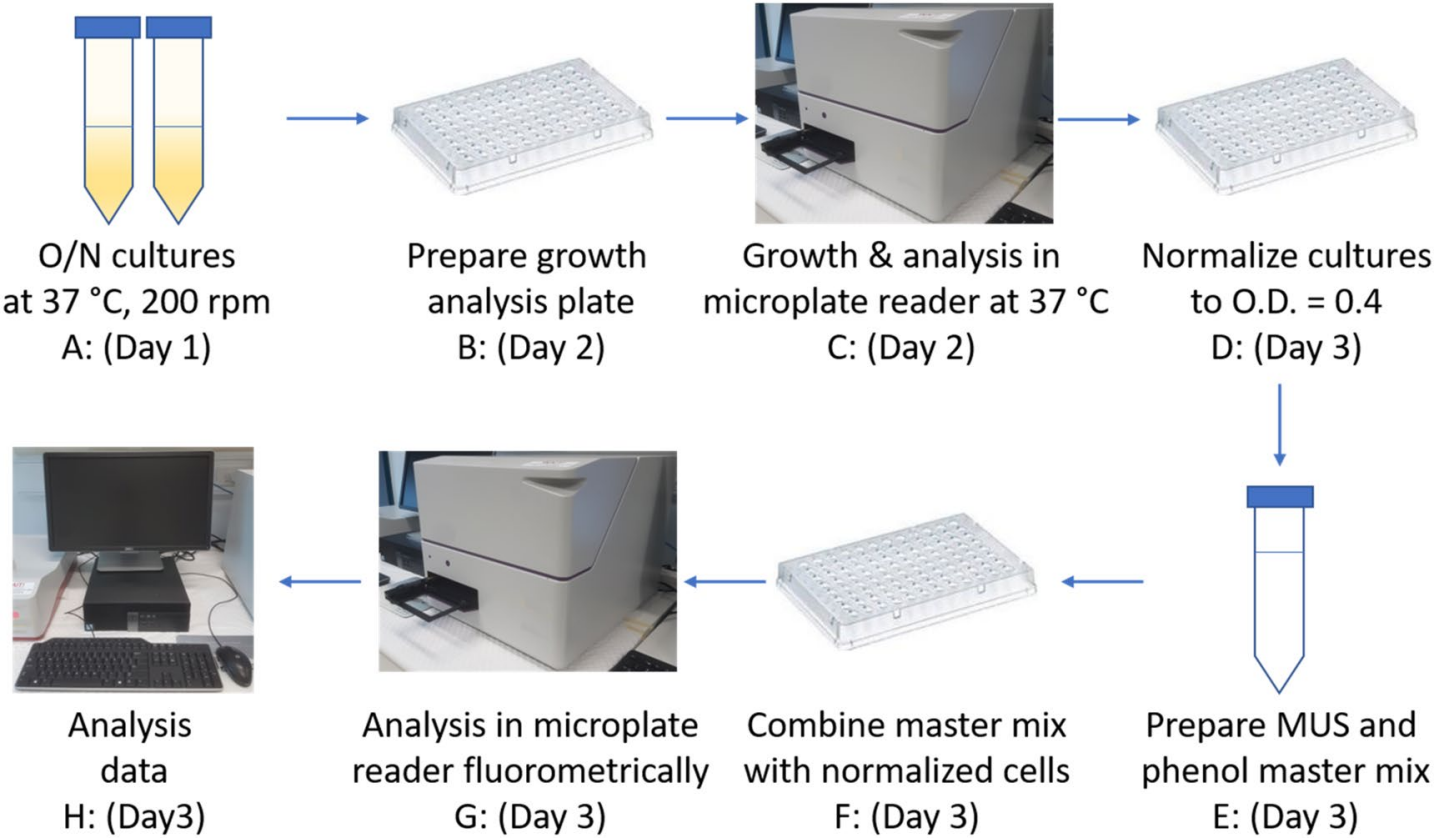
Overview of cell-based ASST sulfotransferase assay. (**A**) Bacterial strains were cultured in LB-Lennox media overnight at 37 °C with aeration (200 rpm). (**B**) Growth analysis plates were prepared by subculturing the O/N cultures from (**A**) into a 96-well plate containing the test inhibitors (akin to preparing an MIC challenge plate). (**C**) Growth plates were incubated at 37 °C, 300 rpm, in a microplate reader programmed to take O.D. 600 nm readings every 15 min for 15 h. (**D**) Growth plate cultures were transferred in a fresh 96-well plate with each culture well normalised at an O.D. 600 nm of 0.4. (**E**) A master mix containing 4 µL of phenol (50 mM), 10 µL of MUS (10 mM), and 126 µL of LB-Lennox per reaction well was prepared. (**F**) The reaction master mix (140 µL) was added to normalized cultures (60 µL) and mixed. (**G**) Fluorescence at 450–480 nm was immediately monitored in a plate reader (BMG, Australia) with measurements obtained every 5 min for up to 90 min. (**H**): Data acquisition and analysis.

Bacterial strains were cultured in LB media, supplemented with antibiotics as appropriate, at 37 °C overnight with shaking at 200 rpm. Overnight cultures were used as inocula in bacterial growth assays (step 1) conducted in a 96-well plate by preparing two-fold serial dilutions of F1, F2 or F4 inhibitor compound or DMSO (vehicle control) at twice the desired final concentration in LB-Lennox medium (100 µL final volume). Each well was then inoculated with 100 µL of 1 × 10^7^ CFU/mL inoculum, to give a total well volume of 200 µL and a final cell concentration of 5 × 10^6^ CFU/mL. The growth analysis plate was covered with a breathable sealing membrane (Breathe-Easy sealing membrane, Sigma, Australia), and incubated at 37 °C for 15 h with shaking (300 rpm) in a CLARIOstar plate reader (BMG, Australia) programmed to obtained O.D. 600 nm measurements every 15 min over the 15-h period. At the end of the culture period, each well was normalized to an O.D. 600 nm of 0.4 (~ 3.5 × 10^8^ CFU/mL) in a fresh 96-well plate (step 2), to ensure that each well contained an equal number of cells. Wells were then supplemented with potassium 4-methylumbelliferyl sulfate (MUS, Sigma, Castle Hill, Australia) (0.5 mM final concentration), and phenol (Sigma, Castle Hill, Australia) (1 mM final concentration) and sulfotransferase activity was monitored immediately in a CLARIOstar plate reader (BMG, Australia) by measuring fluorescence emitted at 450–480 nm (excitation wavelength at 360–380 nm) and measurements acquired every 5 min over a 60–90 min time period (Fig. 8). MUS and other similar substrates are typically used in the mM range when examining ASST function where whole cells or whole cell lysates are used^28,39,40,44^. In our analysis, we found 0.5 mM of MUS and 1 mM of phenol gave the most consistent results (data not shown). Instrument data were normalized (with DMSO vehicle control set at 100%) and analysed using GraphPad Prism 8. The F1 dose–response curve was generated using fluorescence data from the 40-min time point, and the corresponding IC_50_ value was calculated by applying a non-linear regression (curve fit) Y = 100/(1 + 10^((LogIC50-X) × HillSlope))).

### Method and formula for calculating Z′-factor^62^

The Z´-factor was calculated using the formula below and positive and negative genetic controls, CFT073/pASST and CFT073/pSU2718, respectively. The mean Z´-factor was calculated across the 10–60 min time range (Fig. 2A), and positive and negative control values were obtained from two separate experiments each consisting of 4 biological and 3 technical replicates.

$${\text{Z}}^{\prime}{\text{ - Factor}} = 1 - \frac{{3 \times {\text{std}}\left( {{\text{Positive}} \;{\text{control}}} \right) + 3 \times {\text{std}}\left( {{\text{Negative}} \;{\text{control}}} \right)}}{{\left| {{\text{mean}}\left( {{\text{Positive }}\;{\text{control}}} \right) - {\text{mean}}\left( {{\text{Negative}}\; {\text{control}}} \right)} \right|}}$$
